# Hypoxic colorectal cancer‐derived extracellular vesicles deliver microRNA‐361‐3p to facilitate cell proliferation by targeting TRAF3 via the noncanonical NF‐κB pathways

**DOI:** 10.1002/ctm2.349

**Published:** 2021-03-17

**Authors:** Jie Li, Peng Yang, Fangyu Chen, Yuqian Tan, Changzhi Huang, Hengyang Shen, Chaofan Peng, Yifei Feng, Yueming Sun

**Affiliations:** ^1^ Department of Colorectal Surgery The First Affiliated Hospital of Nanjing Medical University Nanjing China; ^2^ Department of Radiation Oncology The First Affiliated Hospital of Nanjing Medical University Nanjing China

**Keywords:** colorectal cancer, extracellular vesicles, hypoxia, miR‐361‐3p, noncanonical NF‐κB

## Abstract

**Background:**

Hypoxic tumour microenvironment (TME) is a key regulator in cancer progression. However, the communications between hypoxic cells and other components in TME during colorectal cancer (CRC) progression via extracellular vesicles (EVs) remain unclear.

**Methods:**

High‐throughput sequencing was employed to detect aberrantly expressed microRNAs (miRNAs) in hypoxic EVs. Quantitative real‐time PCR was used to confirm and screen preliminarily candidate miRNAs. The effects of EVs derived from hypoxia (<1% O_2_) and miR‐361‐3p on CRC growth were assessed using CCK‐8 assays, colony formation assays, EdU assays, flow cytometric assays and mouse xenograft. Then, the specific mechanisms of miR‐361‐3p were investigated by RNA immunoprecipitation, luciferase reporter assay, Western blot, chromatin immunoprecipitation, immunohistochemistry and rescue experiments.

**Results:**

The level of miR‐361‐3p expression was remarkably elevated in hypoxic EVs and can be transferred to CRC cells. Functional experiments exhibited that hypoxic EVs facilitated cell growth and suppressed cell apoptosis by transferring miR‐361‐3p of CRC. Hypoxia‐inducible factor‐1α induced the elevation of miR‐361‐3p levels in hypoxic EVs. Upregulated miR‐361‐3p in CRC inhibited cell apoptosis and facilitated cell growth by directly targeting TNF receptor‐associated factor 3, which consequently activated the noncanonical NF‐κB pathway. Moreover, the high expression of circulating exosomal miR‐361‐3p was correlated to worse prognosis of CRC patients.

**Conclusions:**

Altogether, the abnormality of exosomal miR‐361‐3p derived from hypoxia acts vital roles in the regulation of CRC growth and apoptosis and can be an emerging prognostic biomarker and a therapeutic target for CRC patients.

## Graphical abstract

Hypoxic colorectal cancer‐derived extracellular vesicles deliver microRNA‐361‐3p to facilitate cell proliferation by targeting TRAF3 via the noncanonical NF‐κB pathways. Hypoxia‐inducible factor‐1α induced the elevation of miR‐361‐3p levels in hypoxic EVs. Moreover, the high level of exosomal miR‐361‐3p in the serum from CRC patients was correlated to poor prognosis.

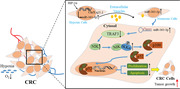

**Abbreviations**
CCK‐8: Cell Counting Kit‐8
CHIP: chromatin immunoprecipitation
CRC: colorectal cancer
DFS: disease‐free survival
EdU: 5‐ethynyl‐2′‐deoxyuridine
EVs: extracellular vesicles
FISH: fluorescence in situ hybridization
HIF‐1α: hypoxia‐inducible factor 1 subunit alpha
IHC: immunohistochemistry
NC: negative control
OS: overall survival rate
qRT‐PCR: quantitative real‐time PCR
RIP: RNA immunoprecipitation
TME: tumour microenvironment
TRAF3: TNF receptor‐associated factor 3

## 1 BACKGROUND

Colorectal cancer (CRC) is the third burdensome malignancy planetwide, with >1,800,000 diagnosed CRC patients and 881 thousand deaths evaluated to occur in 2018.^1^ Despite the substantial progress and development in diagnosis and therapy, the incidence and mortality of CRC remain at high levels.^2^ The main reason for this phenomenon is the limited understanding of the pathogenesis and progression of CRC. Thus, studies should focus on gaining insights into the biological mechanisms of CRC for the improvement of these dismal outcomes.

Hypoxia is a typical feature of almost all human solid tumours; the faster growth of tumour cells than tumour vasculature results in relatively hypoxia of the former.^3^ In response to hypoxia, numerous genes related to the oxygen monitoring mechanism, such as hypoxia‐inducible factors (HIFs), will be activated in the cell, which activate a set of genes that facilitate tumour growth, angiogenesis and metastasis.^4^, ^5^, ^6^ Current studies on cancer and hypoxia should also focus on the interactive communications between hypoxic cells and other components in the tumour microenvironment (TME) instead of focusing solely on the hypoxic cells themselves. Growing evidence emphasises the role of TME in cancer progression.^7^, ^8^

Extracellular vesicles (EVs) are small (diameter: 30–‐200 nm) dual‐membrane particles and released by various types of cells.^9^ They are considered as extracellular messengers between tumour cells and other microenvironment components and play key roles in tumour progression, metastasis and chemoresistance by transporting signal molecules, containing assorted nucleic acids including microRNAs (miRNAs) and long noncoding RNAs, multiple proteins and lipids.^10^, ^11^, ^12^, ^13^ EVs are involved in communication among cells in the TME.^14^, ^15^ EVs also contain abundant miRNAs and have profound effects on the immune response, chemotherapy resistance and metastatic behaviours in various tumours by delivering miRNAs.^15^, ^16^, ^17^, ^18^ Hence, understanding the functions of exosomal miRNAs derived from hypoxic environment in CRC progression is an important research direction.

In this study, we demonstrated that EVs derived from hypoxic conditions can greatly promote the growth and inhibit the apoptosis of CRC cells. miR‐361‐3p was overexpressed in EVs derived from hypoxic environment, as shown by high‐throughput sequencing and qRT‐PCR.^19^ miR‐361‐3p was transferred to normoxic CRC cells and targeted TNF receptor‐associated factor 3 (TRAF3), which acts as a tumour inhibitor that blocks the noncanonical NF‐κB signaling pathway, thus promoting cell growth of CRC. In addition, HIF‐1α can elevate the miR‐361‐3p expression in EVs as a transcription factor. Analysis of clinical data indicated that the exosomal miR‐361‐3p in plasma can be a promising biomarker to forecast the prognosis of CRC patients.

## 2 MATERIALS AND METHODS

### 2.1 Clinical specimens, cell culture and hypoxic treatment

Twenty (20) peripheral blood samples of CRC patients and eighty (80) CRC tissues were obtained from the First Affiliated Hospital of Nanjing Medical University (NJMU). None of 100 CRC patients received any treatments (radiotherapy or neoadjuvant therapy) before operation. The clinical study was ratified by the Ethical Committee of Jiangsu Provincial Hospital.

HCT116 and HT29 were obtained from the Cell Bank of Type Culture Collection of the Chinese Academy of Sciences (Shanghai, China). Both cell lines were cultured in McCoy's 5A medium (Thermo Fisher, Waltham, MA, USA) containing 10% foetal bovine serum (FBS) (Thermo Fisher, USA) or 10% EVs‐free FBS. CRC cells were cultured at 37°C and 5% CO_2_, and the contamination by *mycoplasma* was checked regularly. The hypoxic conditions (<1% O_2_) were induced as previously described.^20^

### 2.2 EV isolation and identification

First, the common FBS was centrifuged at 120,000 g and 4°C for 16 h to obtain EVs‐free FBS. Then, the collections of supernatants after centrifugation were filtered by a 0.22 μm filter (Millipore, Burlington, MA, USA) for subsequent experiments. The cell supernatants collected under normoxic or hypoxic conditions were cultured in medium containing 10% EVs‐free FBS. Next, the blood samples or culture medium were sequentially centrifuged at 500 g for 5 min, 2000 g for 15 min and 12,000 g for half an hour to dispose of floating cells and cell fragments. Finally, the collected supernatants of culture medium or blood samples were ultracentrifuged for 70 min at 120,000 g and 4°C, and the collected sediments at the bottom of the tube were suspended in phosphate‐buffered saline (PBS) and ultracentrifuged at same setting once again. EVs re‐suspended in PBS for ultimate studies.

The representative structure of EVs’ bilayer membranes was identified by transmission electron microscopy. And the diameter distribution and original concentration of EVs were detected by nanoparticle tracking analysis (NTA).^21^ CD63 (Abcam, ab271286), TSG101 (Abcam, ab125011) and CD81 (Abcam, ab109201) were tested as distinctive markers of EVs, and calnexin (Abcam, ab22595) was detected as negative control (NC). The concentration of EVs was analysed by using NTA or BCA Protein Assay Kit (Thermo Fisher, USA).

### 2.3 Labelling of EVs

EVs were labelled by PKH67 (Sigma‐Aldrich, St. Louis, MO, USA) in consistent with manufacturer's protocols. At 1 day after the incubation of PKH67‐labelled‐EVs with CRC cells, DAPI (Beyotime, Shanghai, China) was used for nuclei staining. The co‐incubated cells were recorded by confocal microscopy.

### 2.4 Western blot analysis

Procedures were as described previously.^20^ The detection of protein level was performed by ECL (Millipore) using Bio‐Imaging System. The primary antibody information is listed below: CD63 (Abcam, ab271286), CD81 (Abcam, ab109201), TSG101 (Abcam, ab125011), calnexin (Abcam, ab22595), HIF‐1α (Abcam, ab51608; Cell Signaling Technology, #36169), β‐actin (Abcam, ab8226), TRAF3 (Proteintech, 66310), p27 (Abcam, ab32034), cyclin‐dependent kinase 4 (CDK4) (Abcam, ab108357), cyclin D1 (Abcam, ab16663), Bcl‐2 (Abcam, ab32124), Bax (Abcam, ab32503), NIK (Abcam, ab19144), phosphorylated IKKα/β (Cell Signaling Technology, #2697), caspase‐3 (Cell Signaling Technology, #9662) and NF‐κB2 (Cell Signaling Technology, #3017).

**HIGHLIGHTS**

- This study first confirmed the key role of miR‐361‐3p in hypoxic EVs in promoting proliferation and inhibiting apoptosis of CRC cells.
- miR‐361‐3p promotes CRC progression by targeting the TRAF3‐mediated noncanonical NF‐kB pathway.
- HIF‐1α is the key factor for hypoxic exosomal miR‐361‐3p elevation in CRC cells.

### 2.5 qRT‐PCR and RT‐PCR

Detailed procedures of qRT‐PCR and RT‐PCR were as described previously.^20^ Table S1 lists the primers of mRNAs, miRNAs and internal control. For cell and EV samples, the expression of genes or miRNAs was normalised to those of internal controls (β‐actin or U6) and cel‐miR‐39 (RiboBio, China). 2^− ∆ ∆ CT^ method presented the relative expression of genes or miRNAs.

### 2.6 Cell transfections

GenePharma (Shanghai, China) conducted the vectors of LV2‐hsa‐miR‐361‐3p mimics and inhibitor. The cells transfection was implemented at 40% confluence. GenePharma (Shanghai, China) constructed pcDNA3.1 vectors containing HIF‐1α, sh‐ HIF‐1α, TRAF3, sh‐TRAF3 and the matched NC. RiboBio (Guangzhou, China) conducted siRNAs and miRNA‐mimics.

### 2.7 EdU assay

Procedures was adapted from a previous publication.^22^ Pictures were photographed by using a Nikon microscope.

### 2.8 Luciferase reporter assay

Detailed procedures were consistent with previously described.^20^ GeneScript (Nanjing, China) synthesised the sequences matched to the 3ʹ‐UTR of TRAF3 mRNA and including the wild‐type or mutant miR‐361‐3p binding sequences.

Promoter sequences of wild‐type miR‐361‐3p (WT) and mutant miR‐361‐3p (MUT) were cloned into the pGL3 basic vectors. GenePharma conducted these sequences. Plasmids carrying HIF‐1α or NC and plasmids containing WT or MUT promoters of miR‐361‐3p were co‐transfected to HEK‐293T cells.

### 2.9 RNA immunoprecipitation

Detailed procedures were conducted as previously described.^22^ The RNA immunoprecipitation (RIP) Kit (Millipore) was used to perform the RIP assays. Precipitate was digested, and the co‐immunoprecipitated RNAs were extracted for the further PCR.

### 2.10 Chromatin immunoprecipitation

Chromatin immunoprecipitation (ChIP) assay was conducted as previously described.^23^ Table S1 shows the primer sequences. The JASPAR database was used to perform the prediction of sequence motif.

### 2.11 Cell proliferation assay

After the cells were supplemented with CCK‐8 reagent (Beyotime, China) for 2 h, wavelength of 450 nm was selected to detect the absorbency of cells.^22^

For colony formation assays, procedures were consistent with the descriptions of previous publication.^22^

### 2.12 Fluorescence in‐situ hybridisation and immunohistochemistry

Fluorescence in‐situ hybridisation (FISH) was performed in tissue sections using FISH Kit (RiboBio, Guangzhou, China) and miR‐361‐3p detection probe (GenePharma, Shanghai, China) following the manufacturer's protocol. The DAPI was applied to display of the nuclear. Photographs were snapped by using confocal microscopy (Zeiss, Germany).

Immunohistochemistry (IHC) was conducted as previously described.^20^

### 2.13 Cell cycle and apoptosis analysis

The cells were cleansed twice using PBS after digestion. They were afterwards immobilised with 75% ethanol at ‐20°C for at least 12 h. Subsequently, 75% ethanol was removed, and the cells were washed twice and stained with DNA staining dye (Beyotime, Shanghai, China) at room temperature avoiding light for half an hour. The percentage of cells was detected using BD FACSCanto II (BD Biosciences, San Jose, CA, USA).

Cells were pretreated with 4 μmol H_2_O_2_ for 2 h to stimulate apoptosis before adding the apoptotic reagents. Apoptosis Kit (Vanzyme, China) performed the cell apoptosis assay. Treated cells were lucifugally incubated with 300 μl binding buffer, including 3 μl Annexin V‐ FITC solution and 3 μl PI, for 20 min. BD FACSCanto II was used to analyse the cells.

### 2.14 Mice model

Animal Ethics Committee of NJMU ratified this animal experiment (IACUC‐1901012). Mice (4‐week‐old) were obtained from the Animal Core Facility of NJMU. 1 × 10^6^ cells were first subcutaneously injected. Then, caliper was used to measure tumours every day via the formula: tumour size = (width^2^ × length)/2. The hypoxic EVs, hypoxic EVs and Annexin V, normoxic EVs, PBS, miR‐361‐3p‐EVs or NC‐EVs were injected into the mice via the tail vein once daily for 5 days at the tumour size reached 50 mm^3^. The mice were sacrificed at 14th day after the EVs injection, and the tumours were dissected, photographed and stored in formaldehyde.

### 2.15 TUNEL assay

The TUNEL Kit (Roche, Germany) was used to perform the assay by following manufacturer's protocols. The fluorescence microscope (Leica, Germany) detected the apoptotic cells. TdT was excluded in the reaction mixture as NC.

### 2.16 Statistical analysis

The experiments were conducted thrice independently. Student's *t*‐test, two‐way ANOVA and Kaplan‐Meier analysis performed the data analysis. CRC patients were separated to corresponding group through the median value of tumour size or gene expression. SPSS 22.0 (Chicago, USA) and GraphPad Prism 8.0 (CA, USA) software were used in the analyses, and *p* value < 0.05 was statistically significant. The results in this study were presented as mean ± standard deviation.

## 3 RESULTS

### 3.1 Uptake of hypoxic EVs promotes cell proliferation of CRC in vitro

**FIGURE 1:**
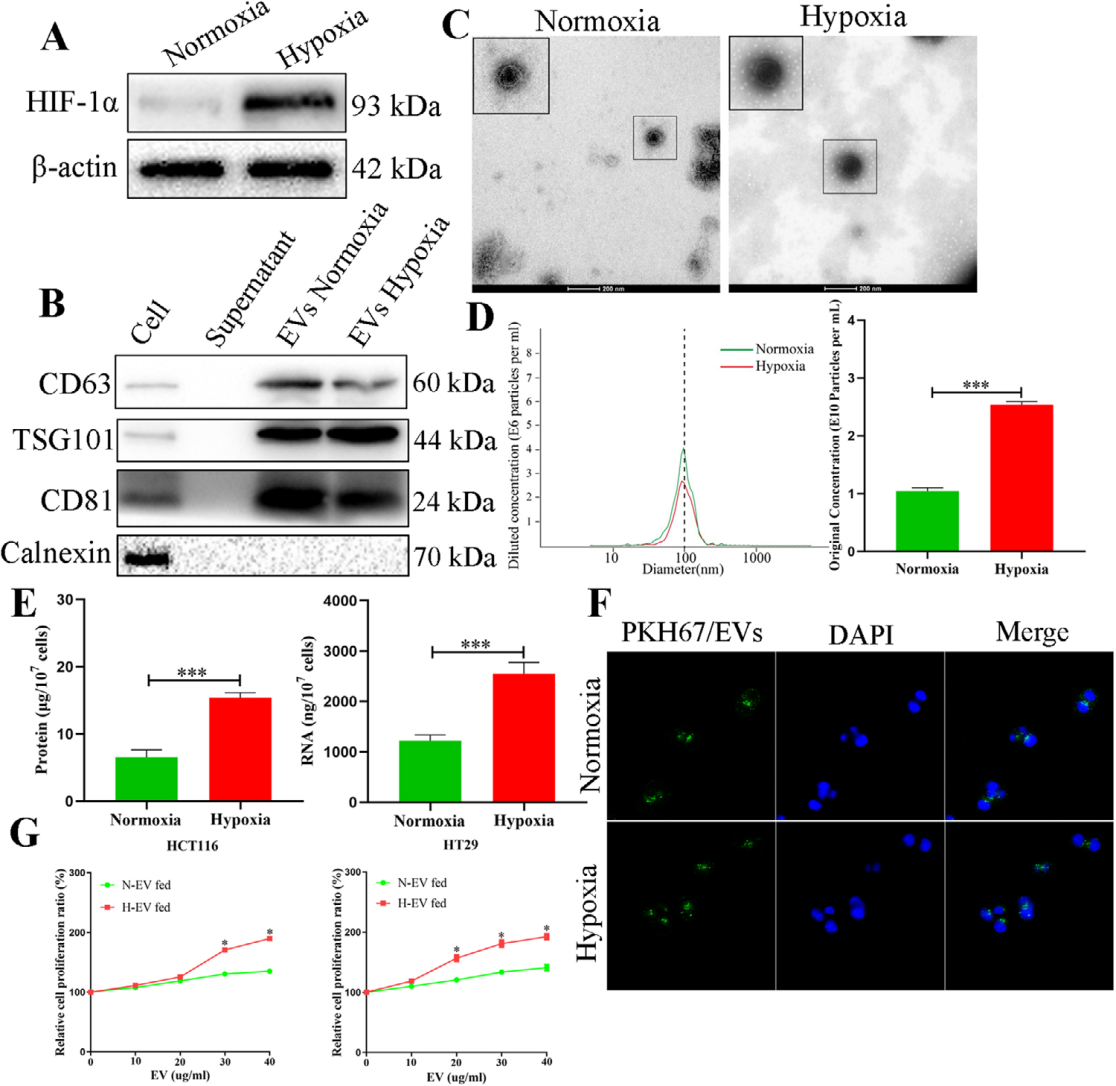
Characterization and roles of extracellular vesicles derived from normoxic and hypoxic CRC cells. (A) The expressions of HIF‐1a in normoxia and hypoxia condition. (B) Western blotting analysis showing extracellular vesicles‐enriched medium with expression of the extracellular vesicles marker of CD63, TSG101 and CD81 and non‐expression of the Calnexin. (C) Extracellular vesicles derived from normoxic and hypoxic CRC cells were analysed under electron microscopy. Scale bar = 200 nm. (D) Nanoparticle tracking analysis was analysed, the size distribution and concentration of extracellular vesicles derived from normoxic and hypoxic CRC cells. (E) EVs derived from CRC cells in a hypoxic environment contain higher levels of protein and RNA in comparison with normoxic conditions. (F) Internalization of extracellular vesicles derived from normoxic and hypoxic CRC cells. Labelled extracellular vesicles (green fluorescent dye, PKH67) were uptake by CRC cells (DAPI‐labelled). (G) CCK8 assay of CRC cells fed with extracellular vesicles derived from normoxic and hypoxic CRC cells at indicated concentrations. Data represent the mean ± SD. Student's *t*‐test was used to determine statistical significance: *p < 0.05 and ***p < 0.001

**FIGURE 2:**
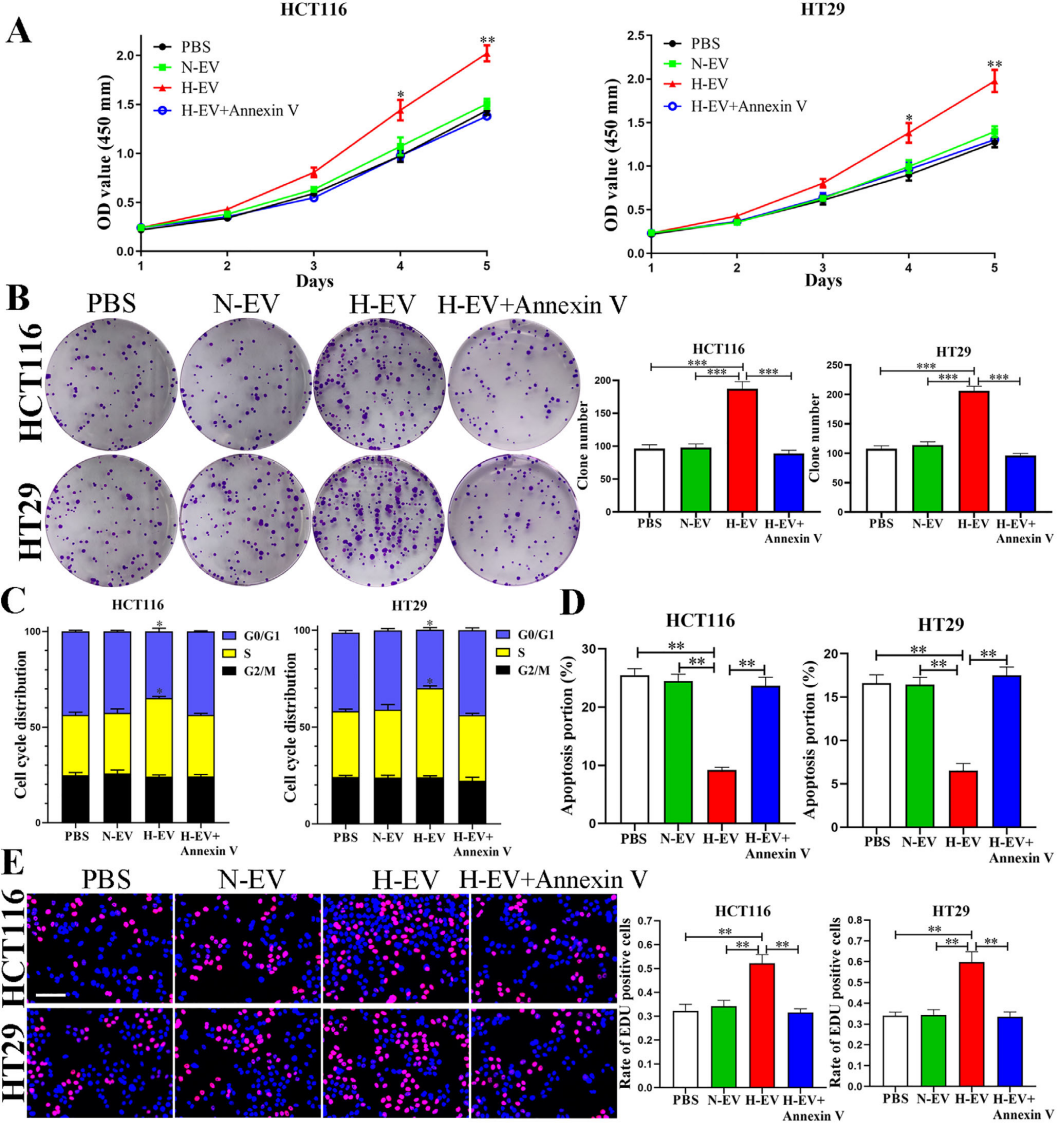
Extracellular vesicles derived from hypoxic CRC cells promoted proliferation of HCT116 and HT29 cells. (A and B) Effects of extracellular vesicles derived from hypoxic CRC cells on proliferation in CRC cell lines were detected by CCK‐8 assay and colony formation. (C and D) Effects of extracellular vesicles derived from hypoxic CRC cells on regulating cell cycle and apoptosis in CRC cell lines. (E) Cell proliferation of CRC cells measured by EDU staining. Scale bar = 200 μm. Data represent the mean ± SD. Two‐way ANOVA and Student's *t*‐test were used to determine statistical significance: *p < 0.05, **p < 0.01 and ***p < 0.001

The effective hypoxia condition was validated by HIF‐1α protein (Figure 1A). The EVs derived from CRC cells expressed specific markers CD63, CD81 and TSG101 but not calnexin (Figure 1B). The typical double‐layer membrane structure of EVs was photographed (Figure 1C). Figure 1D indicated that the diameters of EVs were mainly between 60 nm and 200 nm, and more EVs were produced under hypoxic conditions in the same number of CRC cells. Therefore, the total protein and RNA of hypoxic EVs were higher than those of normoxic EVs derived from the same number of cells, demonstrated that hypoxia may change the contents and quantities of the EVs in CRC (Figure 1E). After 1‐day incubation, EVs‐PKH67 were internalised by recipient cells (Figure 1F). Before performing functional studies, we added normoxic and hypoxic EVs at various concentrations to HCT116 and HT29 cells for 24 h. Hypoxic EVs significantly promoted cell proliferation when their concentration was higher than 30 and 20 μg/ml, respectively (Figure 1G). Thus, we selected 30 μg/ml as the concentration of EVs for the subsequent functional studies. As shown in Figures 2A and 2B, CCK‐8 and colony formation showed that hypoxic EVs could remarkably facilitate the proliferation of CRC cells. In addition, hypoxic EVs promoted cells from G0/G1 to S phase transition and inhibited cell apoptosis of CRC (Figures 2C, 2D, 4A and 4B). Similarly, after internalising of hypoxic EVs, the positive EDU rate of cells was considerably higher than that PBS, normoxic EVs and hypoxic EVs pretreated with Annexin V (an inhibitor of EVs internalization^24^) groups (Figure 2E). These findings demonstrate that EV‐derived hypoxic cells facilitated CRC cell proliferation, induced cell cycle into S phase and inhibited CRC cell apoptosis.

### 3.2 miR‐361‐3p in hypoxic EVs mediates CRC cell proliferation in vitro

**FIGURE 3:**
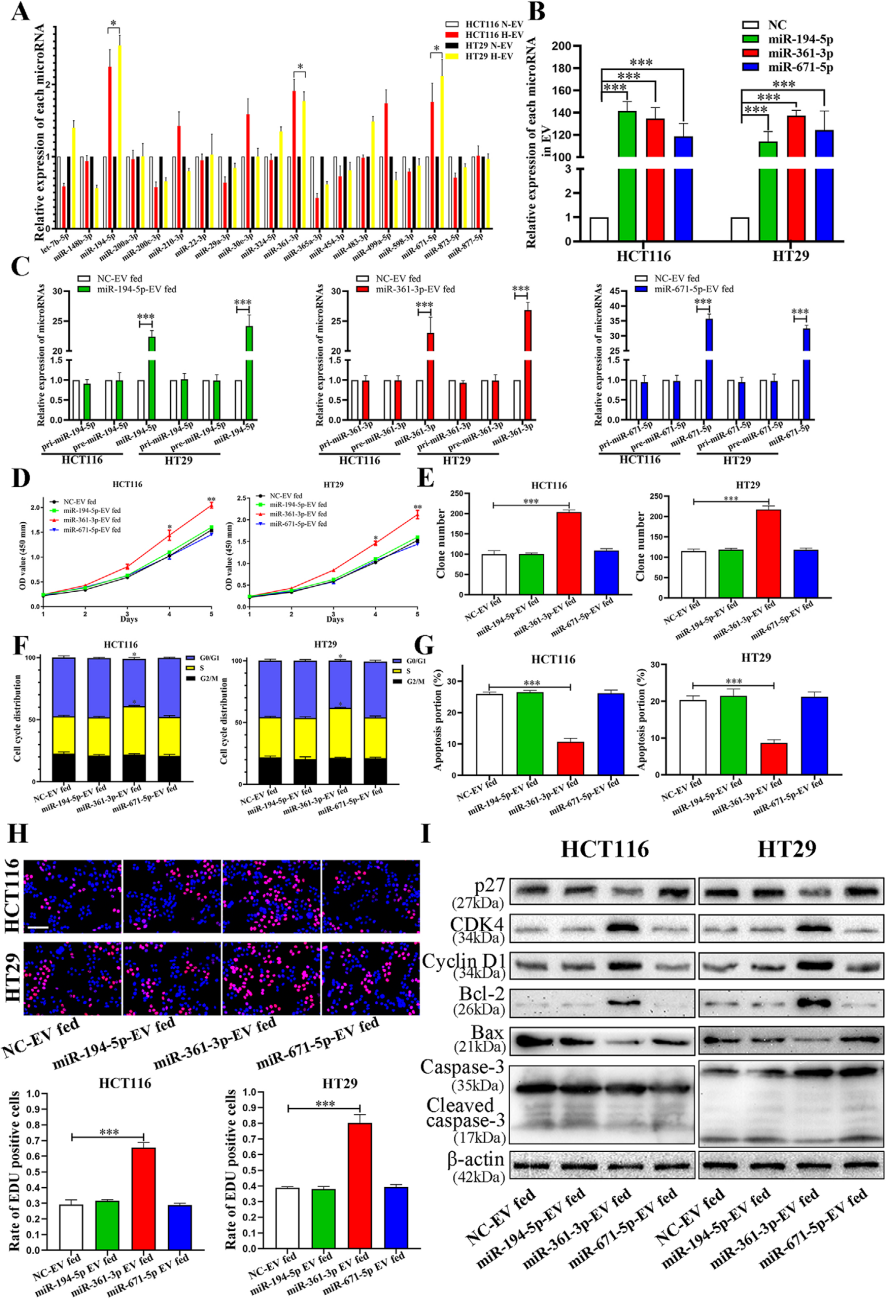
miR‐361‐3p is up‐regulated in hypoxic EVs and transferred by EVs to promote the proliferation of CRC cells. (A) qRT‐PCR was used to detect the expression of microRNAs in normoxic and hypoxic EVs. (B) qRT‐PCR was used to confirm the expression of microRNAs in EVs from cells transfected with miR‐194‐5p‐mimics, miR‐361‐3p‐mimics and miR‐671‐5p‐mimcs, respectively. (C) qRT‐PCR analysis of miR‐194‐5p, miR‐361‐3p and miR‐671‐5p in recipient CRC cells that were treated with NC‐EVs fed, miR‐194‐5p‐EVs fed, miR‐361‐3p‐EVs fed and miR‐671‐5p‐EVs fed. (D and E) Effects of NC‐EVs fed, miR‐194‐5p‐EVs fed, miR‐361‐3p‐EVs fed and miR‐671‐5p‐EVs fed on proliferation in CRC cell lines were detected by CCK‐8 assay and colony formation. (F and G) Effects of NC‐EVs fed, miR‐194‐5p‐EVs fed, miR‐361‐3p‐EVs fed and miR‐671‐5p‐EVs fed on regulating cell cycle and apoptosis in CRC cell lines. (H) Cell proliferation of CRC cells measured by EDU staining. Scale bar = 200 μm. (I) HCT116 and HT29 cells that were fed corresponding EVs were subjected to immunoblotting for p27, CDK4, Cyclin D1, Bcl‐2, Bax, Caspase3 and Cleaved caspase3. Data represent the mean ± SD. Two‐way ANOVA and Student's *t*‐test were used to determine statistical significance: *p < 0.05, **p < 0.01 and ***p < 0.001

**FIGURE 4:**
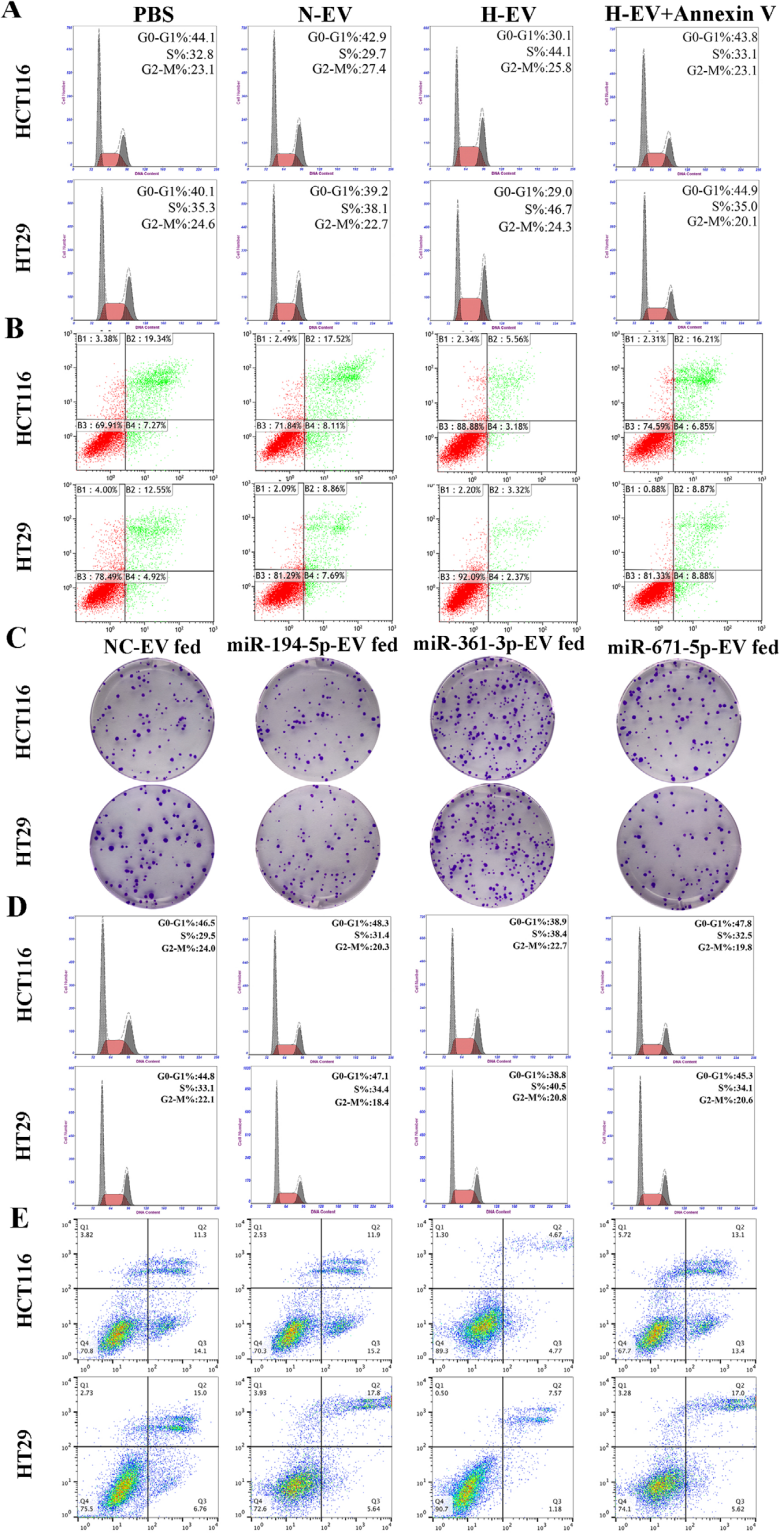
EVs derived from hypoxic CRC cells promoted proliferation of HCT116 and HT29 cells by transferring miR‐361‐3p. (A) Effects of EVs derived from hypoxic CRC cells on regulating cell cycle in CRC cell lines. (B) Effects of hypoxic EVs on regulating apoptosis in CRC cell lines. (C) Effects of NC‐EVs fed, miR‐194‐5p‐EVs fed, miR‐361‐3p‐EVs fed and miR‐671‐5p‐EVs fed on proliferation in CRC cell lines were detected by colony formation. (D) Effects of NC‐EVs fed, miR‐194‐5p‐EVs fed, miR‐361‐3p‐EVs fed and miR‐671‐5p‐EVs fed on regulating cell cycle in CRC cell lines. (E) Effects of corresponding EVs fed on regulating apoptosis in CRC cell lines

Previous study reported that the expression of 19 miRNAs in the EVs secreted by five CRC cell lines under hypoxic condition was notably higher than that of EVs under normoxia via miRNA array.^19^ Subsequently, we verified the expression of these 19 miRNAs in HCT116 and HT29 cell lines. As shown in Figure 3A, miR‐194‐5p, miR‐361‐3p and miR‐671‐5p were both upregulated in EVs secreted by HCT116 and HT29 under hypoxic condition. We further focused on these three miRNAs in the next study. We tested the levels of miR‐194‐5p, miR‐361‐3p and miR‐671‐5p expression in EVs from CRC cell lines transfected to corresponding miRNA‐mimics lentiviruses, respectively (Figure 3B). The incubation with miR‐194‐5p‐EVs significantly increased the intracellular levels of miR‐194‐5p in CRC cells, and the same results appeared in miR‐361‐3p‐EV and miR‐671‐5p‐EV groups (Figure 3C). CCK‐8 and colony formation assay showed that miR‐361‐3p‐EVs remarkably facilitated the growth of CRC cells compared with NC EVs, miR‐194‐5p EVs and miR‐671‐5p EVs (Figures 3D, 3E and 4C). In addition, miR‐361‐3p EVs promoted cells from G0/G1 phase stepped into S phase and inhibited cells apoptosis of CRC (Figures 3F, 3G, 4D and 4E). Similarly, after internalization of miR‐361‐3p EVs, the positive EDU rate of cells was notably higher than that of NC‐EVs, miR‐194‐5p‐EVs and miR‐671‐5p‐EVs groups (Figure 3H). Several critical factors in cell cycle and apoptosis, such as Bcl‐2, Bax, p27, CDK4, CCND1, Caspase‐3 and Cleaved caspase‐3 were evaluated by immunoblot. As shown in Figure 3I, in miR‐361‐3p‐EVs group, Bcl‐2, CDK4 and CCND1 were increased, whereas those of p27, Bax and Cleaved caspase‐3 decreased. No significant changes were observed in caspase‐3 compared with the NC‐EVs, miR‐194‐5p‐EVs and miR‐671‐5p‐EVs groups. Therefore, we clarified the key effects of miR‐361‐3p in hypoxic EVs on promoting cells growth of CRC.

### 3.3 miR‐361‐3p exerts on function by targeting TRAF3

**FIGURE 5:**
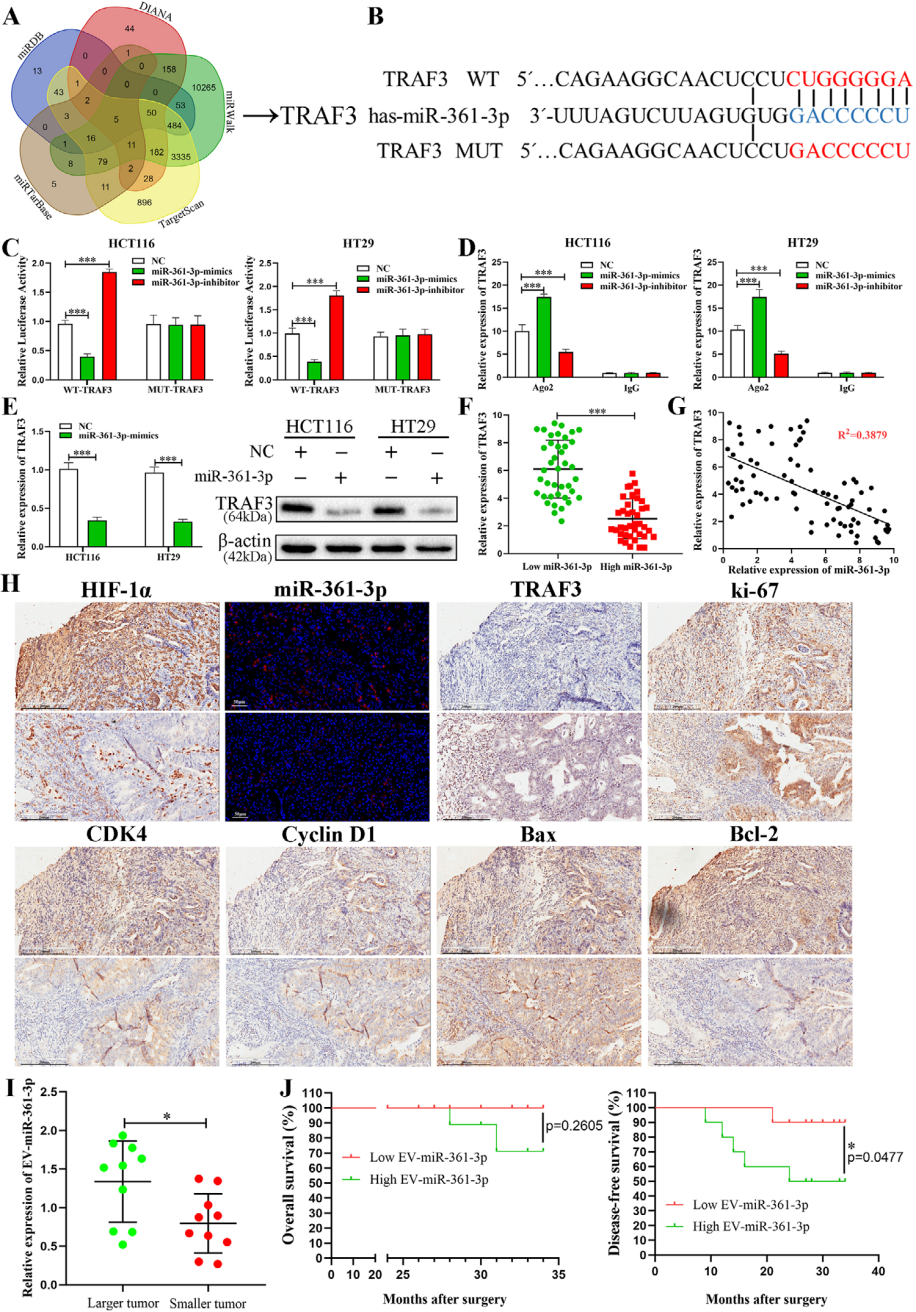
TRAF3 is a direct target of miR‐361‐3p. (A) Venn diagram of predicted miR‐361‐3p targets by five programs (miRDB, DIANA, miRwalk, TargetScan and miRTarBase). (B) The seed regions of miR‐361‐3p, the seed‐recognising sites in the TRAF3 3′ UTR, and the nucleotides mutated in TRAF3 mutant 3′ UTR are shown. (C) Luciferase reporter assay was conducted to verify that miR‐361‐3p bound to the 3′‐UTR region of TRAF3 directly. miR‐361‐3p overexpression significantly suppressed, while miR‐361‐3p loss increased the luciferase activity that carried wild‐type (WT) but not mutant (MUT) 3′‐UTR of TRAF3. (D) RIP assays confirmed the binding status between miR‐361‐3p and TRAF3 in CRC cell lines, respectively. (E) miR‐361‐3p overexpression decreased the level of TRAF3 mRNA and protein in CRC cells. (F) qRT‐PCR analysis indicated that the mRNA expression of TRAF3 in low‐miR‐361‐3p tumours was significantly higher than that in high‐miR‐361‐3p tumours. (G) Pearson's correlation analysis showed the negative correlation of TRAF3 with miR‐361‐3p. (H) Protein levels of HIF‐1α, TRAF3, ki‐67, CDK4, Cyclin D1, Bcl‐2 and Bax in CRC tissues were determined by IHC. Scale bar = 200 μm. FISH was used to detect the miR‐361‐3p in CRC tissues. Scale bar = 50 μm. (I) The relative expression of exosomal miR‐361‐3p in plasma in different tumour size. (J) Comparison of overall survival (OS) and disease‐free survival (DFS) between patients with high expression of exosomal miR‐361‐3p in plasma and low expressing cases in CRC via Kaplan–Meier analysis. Data represent the mean ± SD. Student's *t*‐test was used to determine statistical significance: *p < 0.05 and ***p < 0.001

We used five bioinformatics tools, including miRDB, TargetScan, DIANA, miRwalk and miRTarBase, to seek the potential target of miR‐361‐3p (Figure 5A). A supposed binding site of TRAF3‐3′‐UTR for miR‐361‐3p was discovered (Figure 5B). Analysis of the results of luciferase reporter assay verified that miR‐361‐3p directly targeted to the supposed site of TRAF3 mRNA (Figure 5C). In cells overexpressed miR‐361‐3p, TRAF3 increased, but it decreased in the repressed miR‐361‐3p group, as presented by RIP (Figure 5D). Furthermore, the data of qRT‐PCR (Figure 5E) and Western blots (Figure 5E) revealed the downregulated TRAF3 expression presented in overexpressed miR‐361‐3p cells. The expression of TRAF3 and its correlation with miR‐361‐3p were verified in CRC tissues. Data analysis of clinical characteristics demonstrated that the level of miR‐361‐3p and TRAF3 expression was relative to the size of the tumour (Table S2). The level of TRAF3 was more elevated in the Low‐miR‐361‐3p CRC tissues compared to High‐miR‐361‐3p CRC tissues (Figure 5F). In addition, TRAF3 was significant negative correlations with miR‐361‐3p in CRC tissues (Figure 5G) as shown by Pearson's correlation analysis. Moreover, the mRNA and protein of TRAF3 in different co‐culture conditions were investigated. The results presented that the hypoxic EVs significantly suppressed the TRAF3 expression in comparison with PBS and normoxic EVs (Figures S1A and S1B). IHC was used to detect the expressions of HIF‐1α, TRAF3, ki‐67, CDK4, CCND1, Bax and Bcl‐2 in the same CRC tissues. In combination with the FISH of miR‐361‐3p in CRC, the data indicated that high expression of HIF‐1α increased miR‐361‐3p, whereas the high level of miR‐361‐3p repressed the expression of TRAF3 and promoted the growth of CRC. The expressions of CDK4, CCND1 and Bcl‐2 increased, whereas the expression of Bax that promoted cells apoptosis decreased (Figure 5H). The contrasting results of miR‐361‐3p, TRAF3, ki‐67, CDK4, CCND1, Bax and Bcl‐2 appeared in CRC tissues with low HIF‐1α expression (Figure 5H). In summary, these results indicate that TRAF3 is directly regulated by miR‐361‐3p.

### 3.4 Upregulation of plasma exosomal miR‐361‐3p correlates with poor prognosis of CRC patients

After determining that hypoxic EVs can promote progression of CRC by transferring miR‐361‐3p, we explored its clinical significance for CRC patients. We analysed the exosomal miR‐361‐3p levels in 20 preoperative plasma specimen of CRC patients. Exosomal miR‐361‐3p was increased in serum from larger tumours (3.8 cm as the median value) in CRC patients (Figure 5I). Although no significance was observed in the overall survival between the two groups, the high‐exosomal‐miR‐361‐3p group exhibited substantially worse disease‐free survival in 3 years (Figure 5J). Thus, the levels of exosomal miR‐361‐3p in serum can forecast the prognosis of CRC patients.

### 3.5 miR‐361‐3p promotes proliferation by regulating TRAF3

**FIGURE 6:**
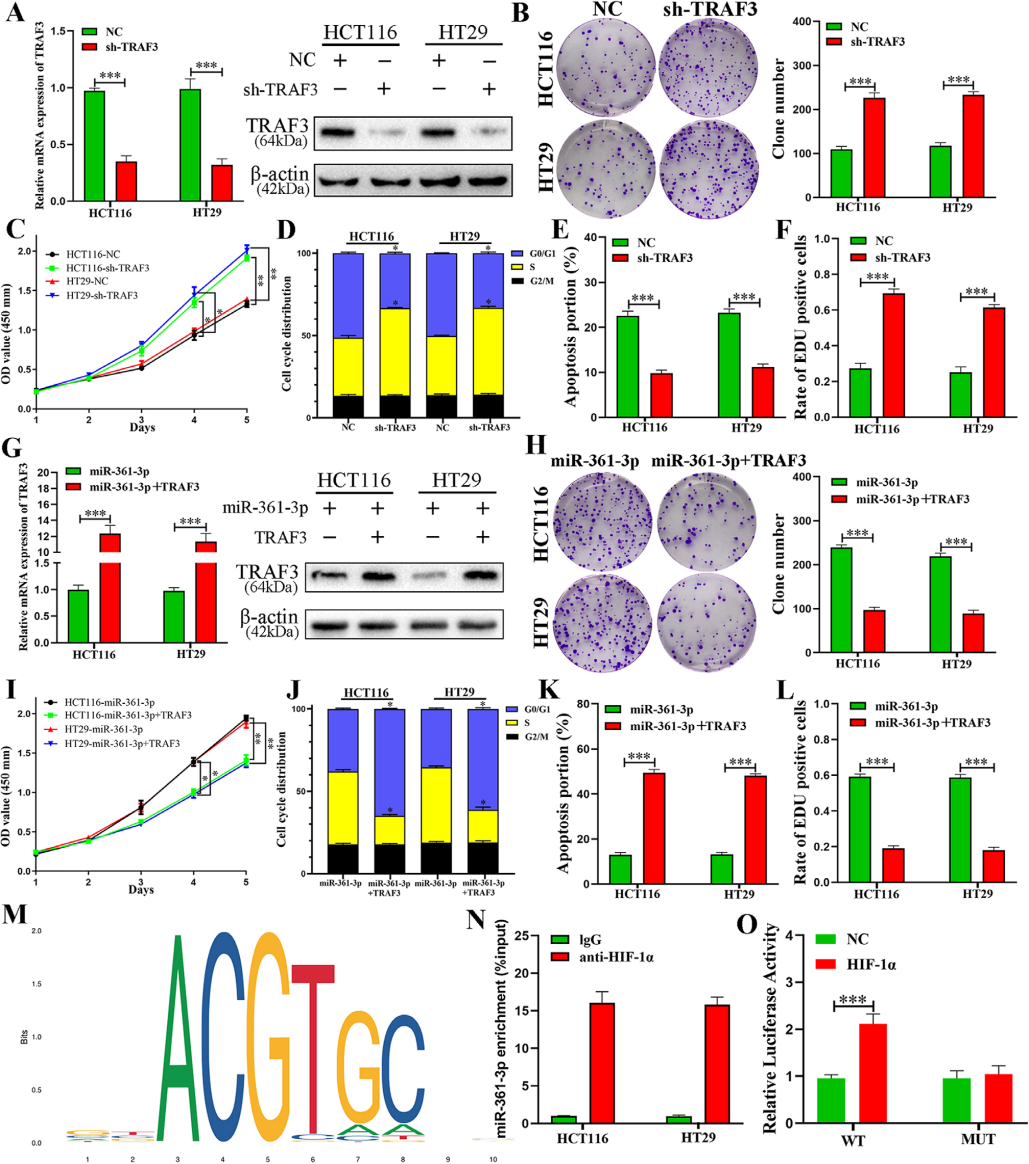
miR‐361‐3p promotes proliferation by targeting TRAF3. (A) Expression of TRAF3 mRNA and protein was verified in transfected CRC cell lines by qRT‐PCR and western blot. (B and C) Effects of NC and sh‐TRAF3 on proliferation in CRC cell lines were detected by CCK‐8 assay and colony formation. (D and E) Effects of NC and sh‐TRAF3 on regulating cell cycle and apoptosis in CRC cell lines. (F) Cell proliferation of CRC cells measured by EDU staining. (G) Expression of TRAF3 was confirmed by qRT‐PCR and western blot in co‐transfected CRC cell lines. (H and I) Effects of miR‐361‐3p+control and miR‐361‐3p+TRAF3 on proliferation in CRC cell lines were detected by CCK‐8 assay and colony formation. (J and K) Effects of miR‐361‐3p+control and miR‐361‐3p+TRAF3 on regulating cell cycle and apoptosis in CRC cell lines. (L) Cell proliferation of CRC cells measured by EDU staining. (M) The predicted sequence motif that HIF‐1α binding in the promoter region of miR‐361‐3p using JASPAR database. (N) Chromatin immunoprecipitation and qRT‐PCR were conducted in CRC cells. (O) Relative luciferase activity of HEK‐293T cells co‐transfected with plasmids of wild‐type (WT) miR‐361‐3p promoters or mutant (MUT) miR‐361‐3p promoters and empty vectors (NC) or HIF‐1α vectors. Data represent the mean ± SD from three independent experiments. Two‐way ANOVA and Student's *t*‐test were used to determine statistical significance: *p < 0.05, **p < 0.01 and ***p < 0.001

sh‐NC and sh‐TRAF3 vectors were transfected to cells to investigate the biological roles of TRAF3 interaction with miR‐361‐3p in CRC. Then, TRAF3 was detected by qRT‐PCR and immunoblot (Figure 6A). CCK‐8 and colony assay indicated that the TRAF3 knockdown substantially facilitated cell proliferation (Figures 6B and 6C). In addition, downregulated TRAF3 induced cells to proceed to the S phase and inhibited cell apoptosis of CRC (Figures 6D, 6E, S2A and S2C). EdU assay presented the similar results (Figures 6F andS2E). Then the cells were co‐transfected with miR‐361‐3p and control, and miR‐361‐3p and TRAF3, respectively. TRAF3 was evaluated via Western blot and qRT‐PCR (Figure 6G). The rescue experiments demonstrated that the proliferation facilitated by overexpressed miR‐361‐3p can be counteracted by an upregulated TRAF3 level (Figures 6H and 6I). Overexpressed TRAF3 arrested cell in G0/G1 phase and promoted cells apoptosis of CRC (Figures 6J, 6K, S2B and S2D). EdU assay presented that high expression of TRAF3 inhibited cell proliferation (Figures 6L and S2F). These data reveal that miR‐361‐3p promotes CRC cell proliferation by targeting TRAF3.

### 3.6 Upregulation of miR‐361‐3p in hypoxic EVs is mediated by hypoxia‐induced elevation of HIF‐1α

To explore the mechanisms of upregulation of miR‐361‐3p in hypoxic EVs, we employed the database of PROMO, LASAGNA and JASPAR to study the specific interaction between the promoter sequence of miR‐361‐3p and transcriptional factors. The results revealed that HIF‐1α has specific biding sites with the promoter sequences of miR‐361‐3p (Figure 6M). Moreover, the results of ChIP assay demonstrated that miR‐361‐3p was enriched in the HIF‐1α group in comparison with the IgG group (Figure 6N). And the luciferase activity of the wild‐type promoter of miR‐361‐3p, and not that of mutant promoter of miR‐361‐3p, was remarkably elevated by HIF‐1α (Figures 6O and S2G). In addition, immunoblot assay revealed that HIF‐1α expression levels were remarkably elevated in CRC cells transfected with HIF‐1α vector (Figure S2H). We subsequently verified the knockdown effect of HIF‐1α via Western blot (Figure S2H). qRT‐PCR demonstrated that HIF‐1α knockdown remarkably decreased miR‐361‐3p levels under hypoxic condition (Figure S2I). The above data demonstrate that HIF‐1α plays a vital role in hypoxia‐induced elevation of miR‐361‐3p in hypoxic CRC EVs.

### 3.7 Hypoxic EVs and miR‐361‐3p promote CRC cell proliferation in vivo

**FIGURE 7:**
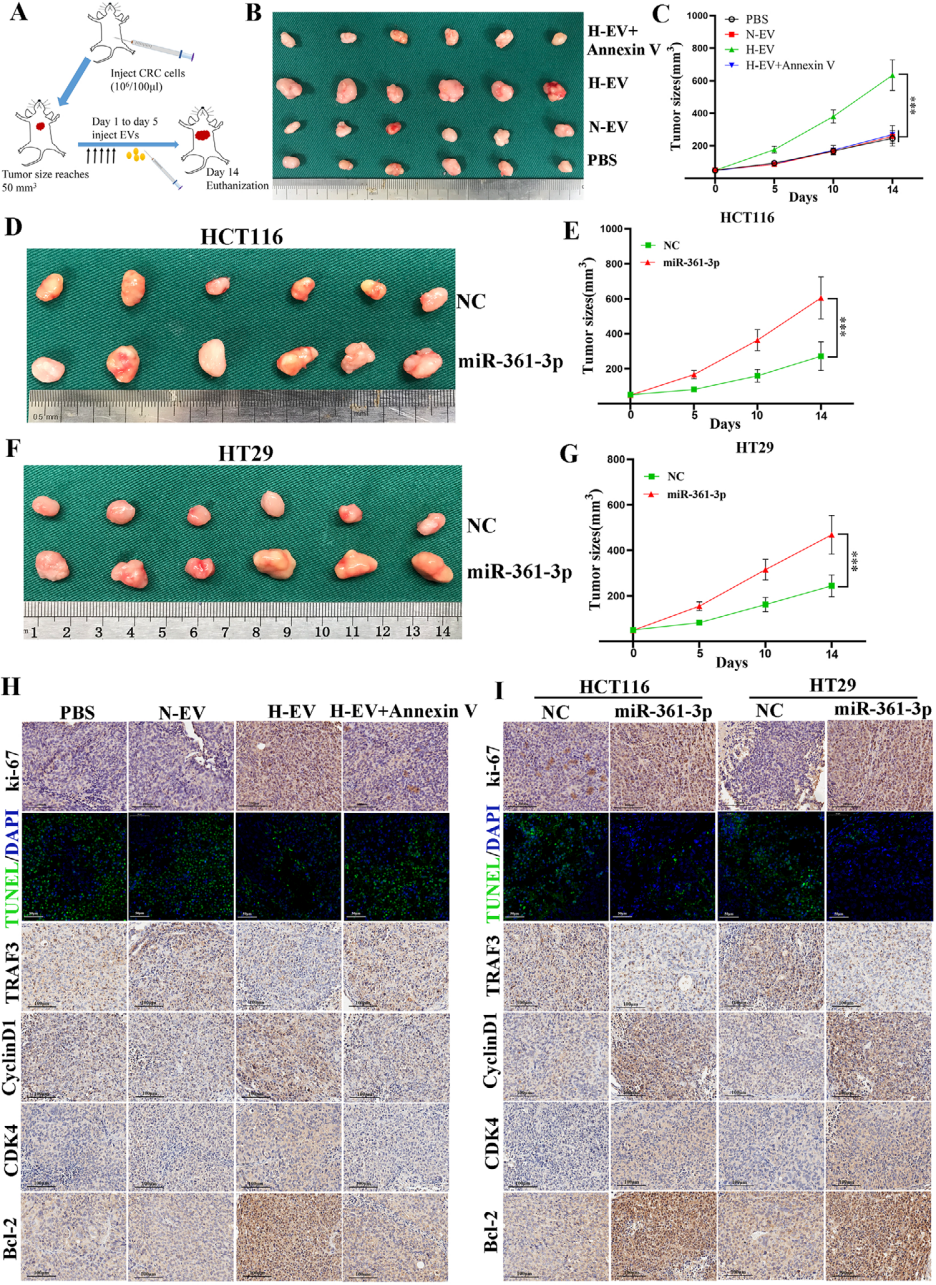
miR‐361‐3p facilitates tumourigenicity of CRC cells in vivo. (A) Schematic model presenting the process to form subcutaneous tumours in mice. (B) Photograph of subcutaneous tumours obtained from nude mice injected with hypoxic EVs+Annexin V, hypoxic EVs, normoxic EVs and PBS. (C) Tumours were observed and recorded by tumour size. (D and F) Photograph of subcutaneous tumours obtained from nude mice injected with NC‐EVs and miR‐361‐3p‐EVs. (E and G) Tumours of different groups were observed and recorded by tumour size. (H) Protein levels of Ki‐67, TRAF3, Cyclin D1, CDK4 and Bcl‐2 in the tumour samples of different groups were determined by IHC. Scale bar = 100 μm. TUNEL was used to detect the apoptosis in the tumour samples. Scale bar = 50 μm. (I) Protein levels of Ki‐67, TRAF3, Cyclin D1, CDK4 and Bcl‐2 in the tumour samples of different groups were determined by IHC. Scale bar = 100 μm. TUNEL was used to detect the apoptosis in the tumour samples. Scale bar = 50 μm. Data represent the mean ± SD. Two‐way ANOVA was used to determine statistical significance: ***p < 0.001

To confirm the effects of hypoxic EVs and miR‐361‐3p on the tumourigenicity of CRC in vivo, we conducted a xenograft model (Figure 7A) by subcutaneously injecting HCT116 and HT29 cells into the flank of mice. When the tumour size reached 50 mm^3^, hypoxic EVs, hypoxic EVs and Annexin V, normoxic EVs, PBS, miR‐361‐3p‐EVs or NC‐EVs were injected into the mice via the tail vein once daily for 5 days. The hypoxic EV‐injected group generated the largest tumours, whereas no significance was observed in hypoxic EVs and Annexin V, normoxic EVs and PBS groups (Figures 7B and 7C). From the IHC of ki‐67, TRAF3, Cyclin D1, CDK4 and Bcl‐2 and TUNEL assay (Figure 7H), the results indicate that hypoxic EVs can facilitate growth by repressing the TRAF3 and promoting cell cycle transition (G0/G1 to S) and inhibit the apoptosis of CRC cells by elevating the Bcl‐2 in vivo. Similarly, tumour growth was significantly boosted by miR‐361‐3p‐EVs injected in comparison with NC‐EVs group (Figures 7D‐7G). The expression of ki‐67, cyclin D1, CDK4 and Bcl‐2 in miR‐361‐3p group was higher than that in NC group, whereas less TRAF3 expression and apoptotic cells were observed in the miR‐361‐3p group (Figure 7I). These findings demonstrate that hypoxic EVs and miR‐361‐3p can promote the growth and inhibit the apoptosis of CRC cells in vivo.

### 3.8 Key role of noncanonical NF‐κB signaling for the function of miR‐361‐3p in CRC

**FIGURE 8:**
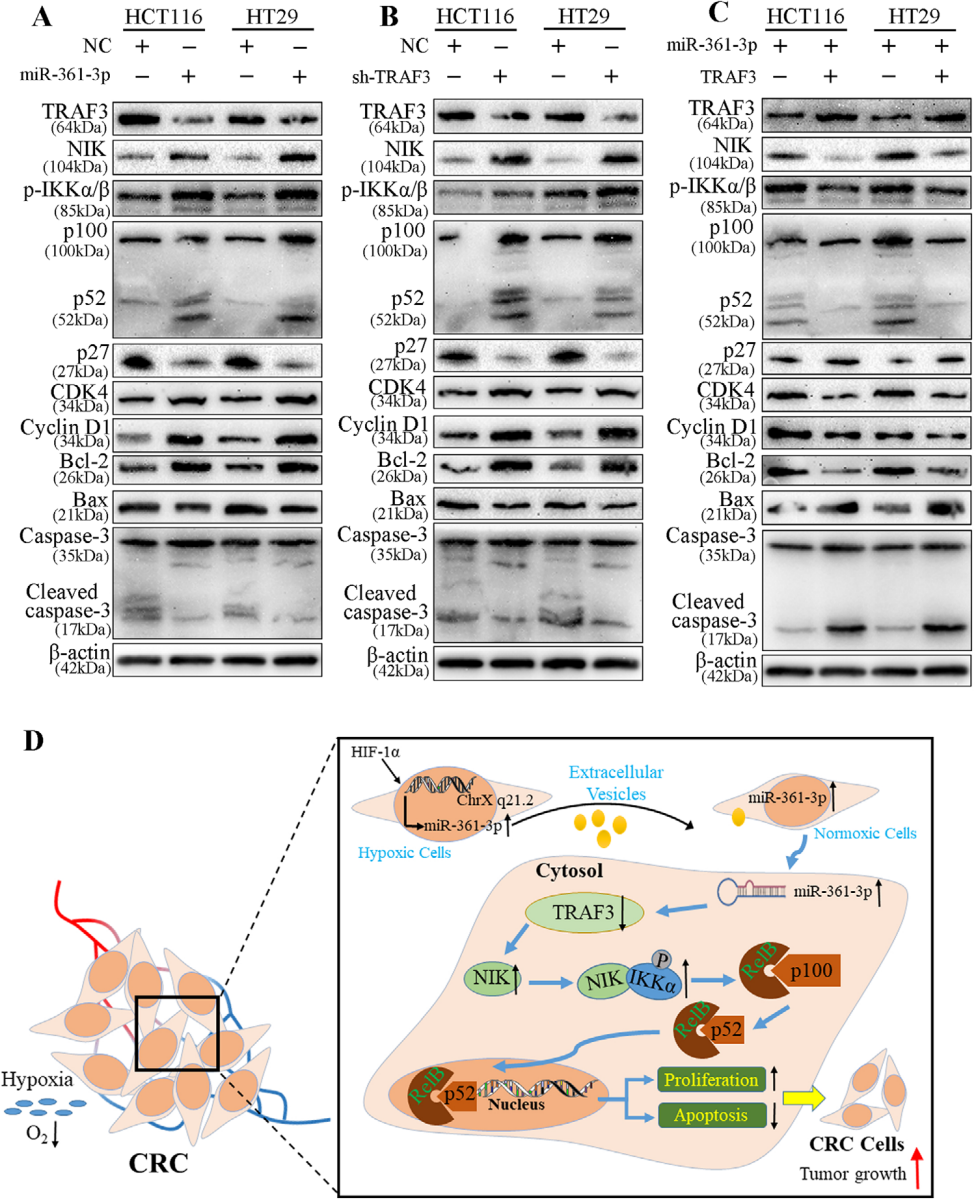
miR‐361‐3p regulates CRC growth via the noncanonical NF‐κB pathways. (A) Immunoblot analysis of TRAF3, NIK, p‐IKKα/β, p100, p52, p27, CDK4, Cyclin D1, Bcl‐2, Bax, Caspase3, Cleaved caspase3 and β‐actin in CRC cells transfected with empty vector, miR‐361‐3p. (B) Immunoblot analysis of abovementioned proteins in CRC cells transfected with sh‐NC, sh‐TRAF3. (C) Immunoblot analysis of abovementioned proteins in CRC cells transfected miR‐361‐3p and co‐transfected groups of miR‐361‐3p and TRAF3. (D) Proposed model of miR‐361‐3p‐promoted proliferation via targeting TRAF3‐activated the noncanonical NF‐kB pathway in CRC cells

TRAF3 is associated with the noncanonical NF‐κB signaling pathway.^25^ To study the biofunction of TRAF3 in CRC, we employed Western blot to detect proteins in the noncanonical NF‐κB signaling. The overexpressed miR‐361‐3p significantly elevated the levels of NIK, phosphorylated IKKα/β, p52, CDK4, CCND1 and Bcl‐2 proteins and decreased TRAF3, p27, Bax and Cleaved caspase 3 proteins (Figure 8A). The data present that upregulated miR‐361‐3p accumulated NIK and phosphorylation‐IKKα/β proteins, thereby activating the noncanonical NF‐κB2 signaling pathway, facilitating proliferation and inhibiting apoptosis in CRC cells. The protein results of TRAF3 knockdown group were similar to those of upregulated miR‐361‐3p treatment (Figure 8B). In the co‐transfected group, the miR‐361‐3p‐promoted effect was rescued by TRAF3 overexpression (Figure 8C). Collectively, the abovementioned results confirm that the hypoxic EVs/miR‐361‐3p/TRAF3 axis regulates CRC growth via the noncanonical NF‐κB pathway (Figure 8D).

## 4 DISCUSSION

Hypoxic environment is an important feature of solid tumours and closely connected to angiogenesis, metastasis, metabolism, proliferation and worse prognosis of cancer.^26^, ^27^ Tumour cells participate in the progression of malignant development of cancer by releasing EVs,^28^ whereas hypoxia is proposed to promote the release of EVs from tumour cells.^29^, ^30^ Focusing on hypoxic cells as drivers of CRC progression, we aimed to investigate the mechanism underlying hypoxia‐mediated development of CRC by intercellular communications. EVs are wrapped in lipid bilayers, which contain a large amount of materials, including proteins and nucleic acids, especially abundant of miRNA. In the present study, EVs were extracted from CRC cells under hypoxic and normoxic conditions. We verified the conclusions of previous reports that hypoxia promoted EV secretions of cancer cells by using NTA and BCA assays.^31^, ^32^ Then, a series of experiments demonstrated that incubation with hypoxic EVs facilitated growth and inhibited apoptosis of CRC cells in vivo and in vitro. Previous studies have confirmed that hypoxic EVs containing miRNAs play a vital role in cancer biology through cell‐to‐cell communications.^10^, ^16^, ^28^, ^33^ However, these studies exploring the roles of hypoxic EVs in cancer are often limited by one or more of the following factors: the screened miRNAs were not validated in clinical samples, the biomarker roles of miRNAs in plasma EVs were lack or the specific mechanisms of miRNAs derived from hypoxic EVs of promoting cancer progression were unclear. In addition, the biological roles of EVs derived from hypoxic condition in CRC by transmitting miRNAs have not been reported so far. Thus, with the confirmation that hypoxic EVs can facilitate proliferation and accelerate progression of CRC, we detected the underlying mechanisms. High‐throughput sequencing technique was used to detect the differential miRNAs between hypoxic and normoxic EVs of CRC cells.^19^ Next, we verified the 19 miRNAs in hypoxic EVs comparison to normoxic EVs of HCT116 and HT29 cell lines via qRT‐PCR. miR‐361‐3p is the critical factor in hypoxic EVs that promote progression of CRC through the screening of functional experiments.

miR‐361‐3p was identified as both tumour suppressor or promoter in the progression of multicancers, such as pancreatic ductal adenocarcinoma,^34^ cervical cancer,^35^, ^36^, ^37^ thyroid cancer,^38^ retinoblastoma,^39^ gastric cancer^40^ and non‐small cell lung cancer^41^, ^42^; however, no study has reported date concerning its role in CRC or hypoxic EVs. In this study, we first identified the oncogenic role of miR‐361‐3p derived from hypoxic EVs in CRC. Incubation with miR‐361‐3p‐EVs remarkably facilitated the growth and inhibited apoptosis of CRC cells in vivo and in vitro, whereas feed NC‐EVs, miR‐194‐5p‐EVs or miR‐671‐5p‐EVs had no effects on proliferative behaviours of CRC cells. These data present that miR‐361‐3p derived from EVs promotes CRC progression by facilitating proliferation and inhibiting apoptosis.

To explore the mechanisms of miR‐361‐3p in CRC, we used bioinformatics tools to seek the potential targets of miR‐361‐3p. Among these mRNA targets, TRAF3, which is widely reported in assorted cancers, was selected through corroboration by qRT‐PCR and IHC in CRC tissues.^43^, ^44^, ^45^ Then, we demonstrated that miR‐361‐3p exerts its biological function in CRC by targeting TRAF3. First, miR‐361‐3p restrained the level of TRAF3 expression in CRC cells. Secondly, the complementary base sequence of miR‐361‐3p was detected in the TRAF3 mRNA. Both overexpressed and downregulated miR‐361‐3p can significantly change the luciferase activity of WT 3′UTR of TRAF3 but causes no effects on that of MUT 3′UTR of TRAF3. Third, TRAF3 was significant negative correlation with the expression of miR‐361‐3p in CRC tissues. Hence, TRAF3 mediates miR‐361‐3p regulation of CRC cell proliferation and apoptosis. TRAF3 can inhibit the activation of noncanonical NF‐κB signaling pathways by inhibiting the accumulation of NIK in cells, thereby preventing the production of phosphorylated IKKα/β and p52.^46^, ^47^, ^48^ We inspected whether TRAF3 is targeted by miR‐361‐3p to validate whether the former is connected with downregulation of p52 expression. Overexpressed miR‐361‐3p remarkably restrained the level of TRAF3 expression, thereby increasing the level of p52. The overexpressed TRAF3 counteracted the proliferation and anti‐apoptotic phenotype induced by miR‐361‐3p. To confirm whether the activation of noncanonical NF‐κB depends on the repression of TRAF3 induced by miR‐361‐3p, we observed whether the overexpression levels of NIK and p52 in CRC cells induced by upregulated miR‐361‐3p were replicated in the TRAF3 knockdown group.

To fully understand why miR‐361‐3p was raised in hypoxic EVs of CRC, we used bioinformatics tools to predict its upstream transcriptional factors. We set sights on HIF‐1α, which has been widely reported that it can stably express only under hypoxic conditions and meanwhile activate the transcription of genes that were involved in tumour metabolism, cell proliferation and metastasis as a transcriptional factor.^26^, ^49^ Stepwise investigations revealed that HIF‐1α can induce miR‐361‐3p expression by binding to specific sequences of its upstream promoter, and silencing HIF‐1α can counteract hypoxia‐mediated miR‐361‐3p expression. These findings demonstrate that HIF‐1α activates the transcription of miR‐361‐3p.

Whether exosomal miR‐361‐3p is valuable as biomarker for CRC patients should be verified. Exosomal miR‐361‐3p in the plasma of larger CRC tumours is higher than that of smaller tumours. The high level of exosomal miR‐361‐3p in serum is significantly related to the unfavorable prognosis of CRC patients. The above data reveal that exosomal miR‐361‐3p in plasma may be a promising biomarker that can be used to forecast the prognosis of CRC patients.

## 5 CONCLUSIONS

In summary, this study confirmed for the first time the key role of miR‐361‐3p in hypoxic EVs in promoting proliferation and inhibiting apoptosis of CRC cells. The elevated level of exosomal miR‐361‐3p in serum is related to advanced clinical pathology of CRC patients. Moreover, miR‐361‐3p promotes the progression and proliferation by targeting the TRAF3‐mediated noncanonical NF‐kB pathway of CRC cells. The upregulated exosomal miR‐361‐3p in serum is an adverse biomarker for CRC patients’ prognosis. Moreover, HIF‐1α is the key factor for hypoxic exosomal miR‐361‐3p elevation in CRC cells. Altogether, the abnormality of hypoxic exosomal miR‐361‐3p plays a vital role in the modulation of tumour growth and apoptosis and may be a new prognostic biomarker that is expected to become a target for CRC treatment.

## CONFLICT OF INTEREST

All authors declare that there is no conflict of interest.

## AUTHOR CONTRIBUTIONS

Yifei Feng, Jie Li and Yueming Sun generated and conducted this experiment. Jie Li, Peng Yang and Yuqian Tan conducted the functional experiments. Jie Li and FYC performed the western blot assay, wrote the manuscript and conducted the animal experiments. Jie Li, Changzhi Huang, Hengyang Shen and Chaofan Peng interpreted and analysed the data. Yueming Sun and Yifei Feng supervised the experiments, secured funding and interpreted results.

## AVAILABILITY OF DATA AND MATERIALS

The datasets used in the current study are available from the corresponding author on reasonable request.

## Supporting information

Supporting information
**Figure S1 Hypoxic EVs repress the expression of TRAF3**. (A) The mRNA expression of TRAF3 was confirmed by qRT‐PCR in PBS, normoxic EVs fed and hypoxic EVs fed groups of CRC cell lines. (B) Western blots were used to detect the TRAF3 expression in PBS, normoxic EVs fed and hypoxic EVs fed groups of CRC cell lines

Supporting information
**Figure S2 miR‐361‐3p promotes proliferation by targeting TRAF3**. (A and C) Effects of NC and sh‐TRAF3 on regulating cell cycle and apoptosis in CRC cell lines. (B and D) Effects of miR‐361‐3p+control and miR‐361‐3p+TRAF3 on regulating cell cycle and apoptosis in CRC cell lines. (E and F) Effects of NC, sh‐TRAF3, miR‐361‐3p+control and miR‐361‐3p+TRAF3 on cell proliferation of CRC cells measured by EDU staining. Scale bar = 200 μm. (G) Schematic representatives of the pGL3‐vectors of wild‐type miR‐361‐3p promoter or mutant miR‐361‐3p promoter construct. (H) Expression of HIF‐1α protein was verified in transfected CRC cell lines by western blot. (I) Expression of miR‐361‐3p was verified in transfected CRC cell lines, normoxia, hypoxia and hypoxia+sh‐HIF‐1α by qRT‐PCR

Supporting information
**Table S1** The primers for qRT‐PCR, RT‐PCR and CHIP, and short hairpin RNAs sequence

Supporting information
**Table S2** Relevance analysis of miR‐361‐3p and TRAF3 expression in CRC patients
