# Supplementary figures and images for: Hypoxic colorectal cancer‐derived extracellular vesicles deliver microRNA‐361‐3p to facilitate cell proliferation by targeting TRAF3 via the noncanonical NF‐κB pathways
Source: Clin Transl Med. 2021 Mar 17;11(3):e349. doi: 10.1002/ctm2.349 (PMC7967919; doi:10.1002/ctm2.349)

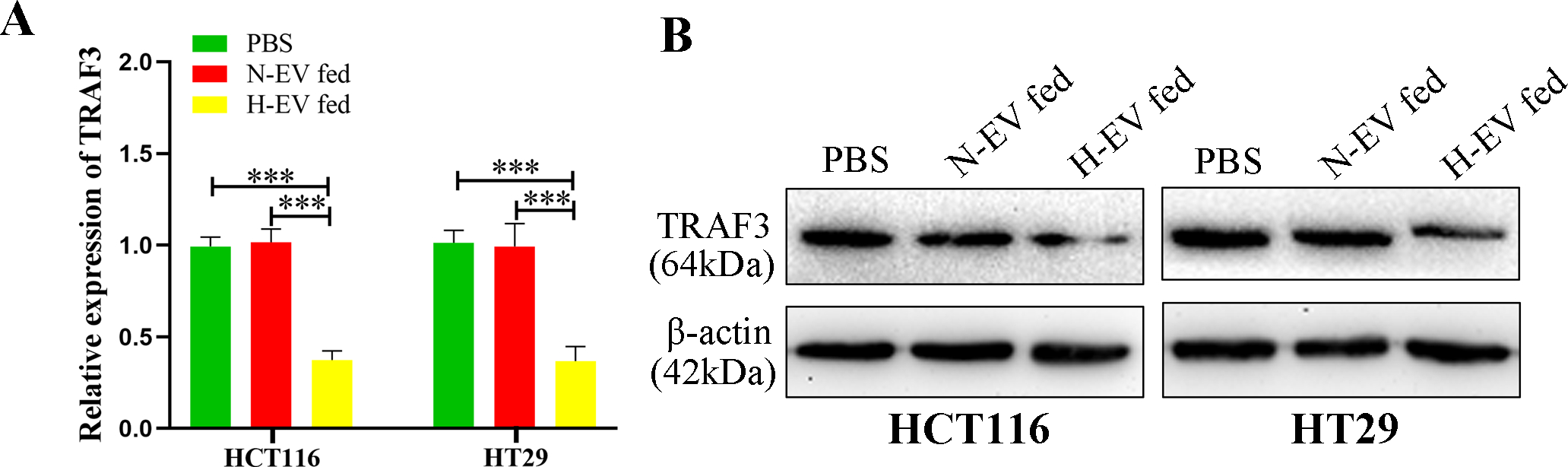

Supplement: Supplementary file 1 — Supporting information Figure S1 Hypoxic EVs repress the expression of TRAF3. (A) The mRNA expression of TRAF3 was confirmed by qRT‐PCR in PBS, normoxic EVs fed and hypoxic EVs fed groups of CRC cell lines. (B) Western blots were used to detect the TRAF3 expression in PBS, normoxic EVs fed and hypoxic EVs fed groups of CRC cell lines [file CTM2-11-e349-s002.tif]

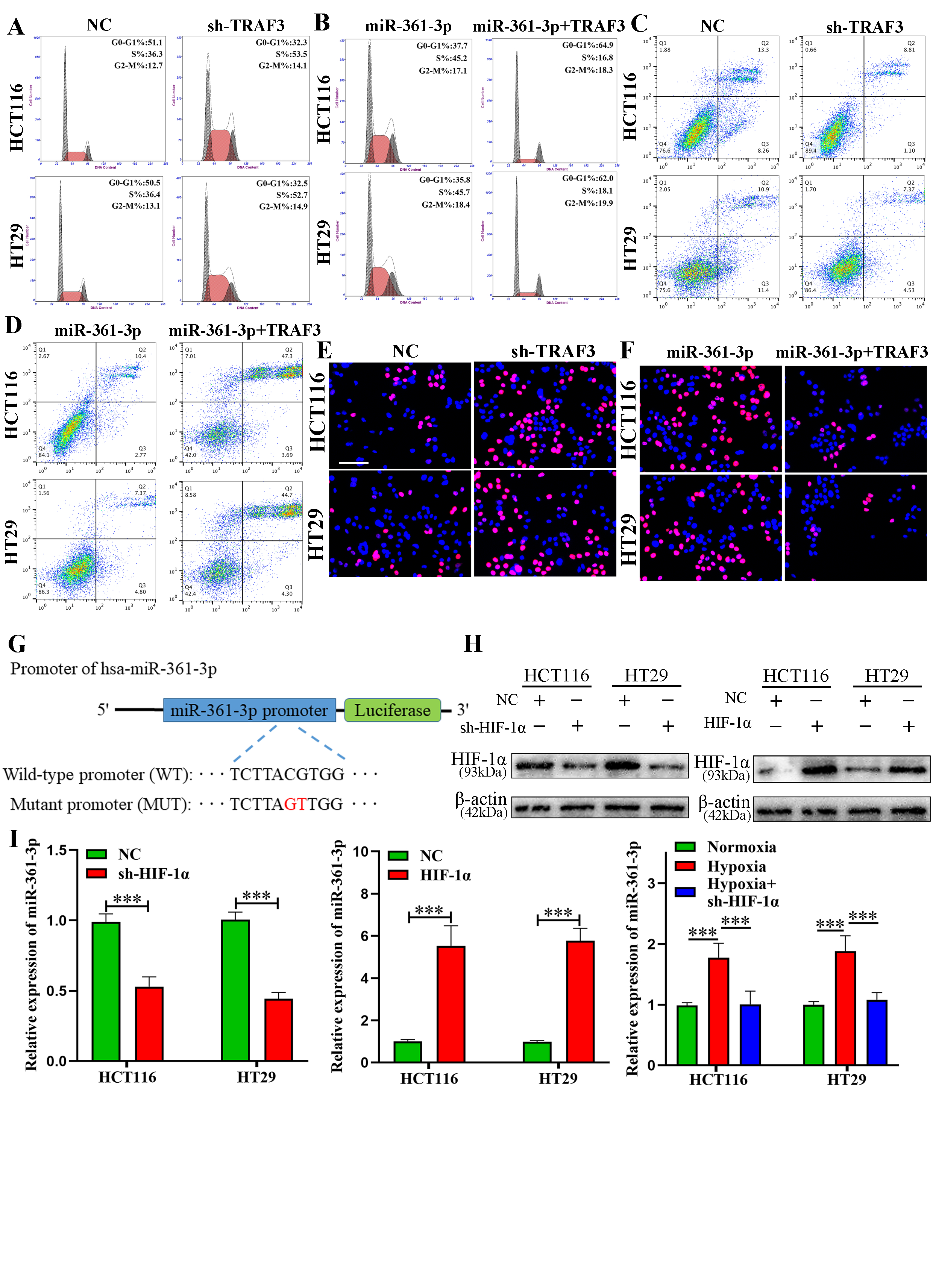

Supplement: Supplementary file 2 — Supporting information Figure S2 miR‐361‐3p promotes proliferation by targeting TRAF3. (A and C) Effects of NC and sh‐TRAF3 on regulating cell cycle and apoptosis in CRC cell lines. (B and D) Effects of miR‐361‐3p+control and miR‐361‐3p+TRAF3 on regulating cell cycle and apoptosis in CRC cell lines. (E and F) Effects of NC, sh‐TRAF3, miR‐361‐3p+control and miR‐361‐3p+TRAF3 on cell proliferation of CRC cells measured by EDU staining. Scale bar = 200 μm. (G) Schematic representatives of the pGL3‐vectors of wild‐type miR‐361‐3p promoter or mutant miR‐361‐3p promoter construct. (H) Expression of HIF‐1α protein was verified in transfected CRC cell lines by western blot. (I) Expression of miR‐361‐3p was verified in transfected CRC cell lines, normoxia, hypoxia and hypoxia+sh‐HIF‐1α by qRT‐PCR [file CTM2-11-e349-s004.tif]
