# Supplementary material for: Hypoxic colorectal cancer‐derived extracellular vesicles deliver microRNA‐361‐3p to facilitate cell proliferation by targeting TRAF3 via the noncanonical NF‐κB pathways
Source: Clin Transl Med. 2021 Mar 17;11(3):e349. doi: 10.1002/ctm2.349 (PMC7967919; doi:10.1002/ctm2.349)
Supplement: Supplementary file 3 — Supporting information Table S1 The primers for qRT‐PCR, RT‐PCR and CHIP, and short hairpin RNAs sequence [file CTM2-11-e349-s003.docx]

**Supplementary Table 1:The primers for qRT-PCR.**

| hsa-TRAF3 | Forward Primer | CAGACTAACCCGCCGCTAAAG |
| --- | --- | --- |
|  | Reverse Primer | GATGCTCTCTTGACACGCTGT |
| hsa-β-actin | Forward Primer | CATGTACGTTGCTATCCAGGC |
|  | Reverse Primer | CTCCTTAATGTCACGCACGAT |
| hsa-U6 | Forward Primer | CTCGCTTCGGCAGCACA |
|  | Reverse Primer | AACGCTTCACGAATTTGCGT |
| hsa-let-7b-5p | Forward Primer | CGGGCTGAGGTAGTAGGTTG |
|  | Reverse Primer | CAGCCACAAAAGAGCACAAT |
| hsa-miR-148b-3p | Forward Primer | CGGGCTCAGTGCATCACAGA |
|  | Reverse Primer | CAGCCACAAAAGAGCACAAT |
| hsa-miR-194-5p | Forward Primer | CGGGCTGTAACAGCAACTCC |
|  | Reverse Primer | CAGCCACAAAAGAGCACAAT |
| hsa-miR-200a-3p | Forward Primer | CGGGCTAACACTGTCTGGTA |
|  | Reverse Primer | CAGCCACAAAAGAGCACAAT |
| hsa-miR-200c-3p | Forward Primer | CGGGCTAATACTGCCGGGTAA |
|  | Reverse Primer | CAGCCACAAAAGAGCACAAT |
| hsa-miR-210-3p | Forward Primer | CGGGCCTGTGCGTGTGACAG |
|  | Reverse Primer | CAGCCACAAAAGAGCACAAT |
| hsa-miR-22-3p | Forward Primer | CGGGCAAGCTGCCAGTTGAA |
|  | Reverse Primer | CAGCCACAAAAGAGCACAAT |
| hsa-miR-29a-3p | Forward Primer | CGGGCTAGCACCATCTGAAA |
|  | Reverse Primer | CAGCCACAAAAGAGCACAAT |
| hsa-miR-30e-3p | Forward Primer | CGGGCCTTTCAGTCGGATGT |
|  | Reverse Primer | CAGCCACAAAAGAGCACAAT |
| hsa-miR-324-5p | Forward Primer | CGGGCCGCATCCCCTAGGGC |
|  | Reverse Primer | CAGCCACAAAAGAGCACAAT |
| hsa-miR-361-3p | Forward Primer | CGGGCTCCCCCAGGTGTGATT |
|  | Reverse Primer | CAGCCACAAAAGAGCACAAT |
| hsa-miR-365a-3p | Forward Primer | CGGGCTAATGCCCCTAAAAA |
|  | Reverse Primer | CAGCCACAAAAGAGCACAAT |
| hsa-miR-454-3p | Forward Primer | CGGGCTAGTGCAATATTGCTT |
|  | Reverse Primer | CAGCCACAAAAGAGCACAAT |
| hsa-miR-483-3p | Forward Primer | CGGGCTCACTCCTCTCCTC |
|  | Reverse Primer | CAGCCACAAAAGAGCACAAT |
| hsa-miR-499a-5p | Forward Primer | CGGGCTTAAGACTTGCAGT |
|  | Reverse Primer | CAGCCACAAAAGAGCACAAT |
| hsa-miR-598-3p | Forward Primer | CGGGCTACGTCATCGTTGTC |
|  | Reverse Primer | CAGCCACAAAAGAGCACAAT |
| hsa-miR-671-5p | Forward Primer | CGGGCAGGAAGCCCTGGAGGG |
|  | Reverse Primer | CAGCCACAAAAGAGCACAAT |
| hsa-miR-873-5p | Forward Primer | CGGGCGCAGGAACTTGTGA |
|  | Reverse Primer | CAGCCACAAAAGAGCACAAT |
| hsa-miR-877-5p | Forward Primer | CGGGCGTAGAGGAGATGG |
|  | Reverse Primer | CAGCCACAAAAGAGCACAAT |

**The primers for RT-PCR**

| hsa-let-7b-5p | CCTGTTGTCTCCAGCCACAAAAGAGCACAATATTTCAGGAGACAACAGGAACCACA |
| --- | --- |
| hsa-miR-148b-3p | CCTGTTGTCTCCAGCCACAAAAGAGCACAATATTTCAGGAGACAACAGGACAAAGT |
| hsa-miR-194-5p | CCTGTTGTCTCCAGCCACAAAAGAGCACAATATTTCAGGAGACAACAGGTCCACAT |
| hsa-miR-200a-3p | CCTGTTGTCTCCAGCCACAAAAGAGCACAATATTTCAGGAGACAACAGGACATCGT |
| hsa-miR-200c-3p | CCTGTTGTCTCCAGCCACAAAAGAGCACAATATTTCAGGAGACAACAGGTCCATCA |
| hsa-miR-210-3p | CCTGTTGTCTCCAGCCACAAAAGAGCACAATATTTCAGGAGACAACAGGTCAGCCG |
| hsa-miR-22-3p | CCTGTTGTCTCCAGCCACAAAAGAGCACAATATTTCAGGAGACAACAGGACAGTTC |
| hsa-miR-29a-3p | CCTGTTGTCTCCAGCCACAAAAGAGCACAATATTTCAGGAGACAACAGGTAACCGA |
| hsa-miR-30e-3p | CCTGTTGTCTCCAGCCACAAAAGAGCACAATATTTCAGGAGACAACAGGGCTGTAA |
| hsa-miR-324-5p | CCTGTTGTCTCCAGCCACAAAAGAGCACAATATTTCAGGAGACAACAGGCACCAAT |
| hsa-miR-361-3p | CCTGTTGTCTCCAGCCACAAAAGAGCACAATATTTCAGGAGACAACAGGAAATCAG |
| hsa-miR-365a-3p | CCTGTTGTCTCCAGCCACAAAAGAGCACAATATTTCAGGAGACAACAGGATAAGGA |
| hsa-miR-454-3p | CCTGTTGTCTCCAGCCACAAAAGAGCACAATATTTCAGGAGACAACAGGACCCTAT |
| hsa-miR-483-3p | CCTGTTGTCTCCAGCCACAAAAGAGCACAATATTTCAGGAGACAACAGGAAGACGG |
| hsa-miR-499a-5p | CCTGTTGTCTCCAGCCACAAAAGAGCACAATATTTCAGGAGACAACAGGAAACATC |
| hsa-miR-598-3p | CCTGTTGTCTCCAGCCACAAAAGAGCACAATATTTCAGGAGACAACAGGTGACGAT |
| hsa-miR-671-5p | CCTGTTGTCTCCAGCCACAAAAGAGCACAATATTTCAGGAGACAACAGGCTCCAGC |
| hsa-miR-873-5p | CCTGTTGTCTCCAGCCACAAAAGAGCACAATATTTCAGGAGACAACAGGAGGAGAC |
| hsa-miR-877-5p | CCTGTTGTCTCCAGCCACAAAAGAGCACAATATTTCAGGAGACAACAGGCCCTGCG |
| hsa-U6 | AACGCTTCACGAATTTGCGT |

**CHIP Primer**

| miR-361-CHIP-166bp | Forward Primer | TCAGCTTGGTCATTGTGTCC |
| --- | --- | --- |
|  | Reverse Primer | GTGCTGCCAGAGTCATGAAA |

**short hairpin RNAs (shRNAs) sequence**

| sh-TRAF3 | sense | GATCCGCGAGAACTCCTCTTTCCCTCGAGGGAAAGAGGAGTTCTCGCAGA |
| --- | --- | --- |
|  | antisense | AGCTTCTGCGAGAACTCCTCTTTCCCTCGAGGGAAAGAGGAGTTCTCGCG |
| sh-HIF-1α | sense | CCGGGGGCCGUUCAAUUUAUGAATTCTCGAGUUCAUAAAUUGAACGGCCCTTTTTTTG |
|  | antisense | AATTCAAAAAGGGCCGUUCAAUUUAUGAATTCTCGAGUUCAUAAAUUGAACGGCCCTT |
