# Supplementary material for: Hypoxic colorectal cancer‐derived extracellular vesicles deliver microRNA‐361‐3p to facilitate cell proliferation by targeting TRAF3 via the noncanonical NF‐κB pathways
Source: Clin Transl Med. 2021 Mar 17;11(3):e349. doi: 10.1002/ctm2.349 (PMC7967919; doi:10.1002/ctm2.349)
Supplement: Supplementary file 4 — Supporting information Table S2 Relevance analysis of miR‐361‐3p and TRAF3 expression in CRC patients [file CTM2-11-e349-s001.docx]

| **Table S2 Relevance analysis of miR-361-3p and TRAF3 expression in CRC patients.** | | | | | | |
| --- | --- | --- | --- | --- | --- | --- |
| **Varible** | **miR-361-3p** | | **P value** | **TRAF3** | | **P value** |
|  | **High** | **Low** |  | **High** | **Low** |  |
| All Cases | 40 | 40 |  | 40 | 40 |  |
| Age (years) | | | | | | |
| <60 | 17 | 16 | 0.8196 | 15 | 18 | 0.4960 |
| ≥60 | 23 | 24 |  | 25 | 22 |  |
| Gender | | | | | | |
| Male | 23 | 22 | 0.8213 | 21 | 24 | 0.4990 |
| Female | 17 | 18 |  | 19 | 16 |  |
| Tumor size (cm) |  | | |  |  |  |
| <5 | 12 | 24 | 0.0070^**^ | 25 | 11 | 0.0017^**^ |
| ≥5 | 28 | 16 |  | 15 | 29 |  |
| TNM staging system |  | | |  |  |  |
| T1 + T2 | 13 | 24 | 0.0136^*^ | 25 | 12 | 0.0036^**^ |
| T3 + T4 | 27 | 16 |  | 15 | 28 |  |
| Tumor stage |  | | |  |  |  |
| Stage I+II | 18 | 23 | 0.2633 | 24 | 17 | 0.1174 |
| Stage III+IV | 22 | 17 |  | 16 | 23 |  |
| Lymph node metastasis |  | | |  |  |  |
| No | 23 | 27 | 0.3557 | 28 | 22 | 0.1659 |
| Yes | 17 | 13 |  | 12 | 18 |  |
| Distant metastasis |  | | |  |  |  |
| No | 31 | 33 | 0.5759 | 34 | 30 | 0.2636 |
| Yes | 9 | 7 |  | 6 | 10 |  |

**P* < 0.05, and ***P* < 0.01.
